# Polydatin protects the respiratory system from PM_2.5_ exposure

**DOI:** 10.1038/srep40030

**Published:** 2017-01-09

**Authors:** Xiao-Dan Yan, Qi-Ming Wang, Cai Tie, Hong-Tao Jin, Yan-Xing Han, Jin-Lan Zhang, Xiao-Ming Yu, Qi Hou, Piao-Piao Zhang, Ai-Ping Wang, Pei-Cheng Zhang, Zhonggao Gao, Jian-Dong Jiang

**Affiliations:** 1 State Key Laboratory of Bioactive Substances and Function of Natural Medicines, Institute of Materia Medica, Chinese Academy of Medical Sciences, Peking Union Medical College, Beijing 100050, China

## Abstract

Atmospheric particle is one of the risk factors for respiratory disease; however, their injury mechanisms are poorly understood, and prevention methods are highly desirable. We constructed artificial PM_2.5_ (aPM_2.5_) particles according to the size and composition of actual PM_2.5_ collected in Beijing. Using these artificial particles, we created an inhalation-injury animal model. These aPM_2.5_ particles simulate the physical and chemical characteristics of the actual PM_2.5_, and inhalation of the aPM_2.5_ in rat results in a time-dependent change in lung suggesting a declined lung function, injury from oxidative stress and inflammation in lung. Thus, this aPM_2.5_-caused injury animal model may mimic that of the pulmonary injury in human exposed to airborne particles. In addition, polydatin (PD), a resveratrol glucoside that is rich in grapes and red wine, was found to significantly decrease the oxidative potential (OP) of aPM_2.5_ *in vitro*. Treating the model rats with PD prevented the lung function decline caused by aPM_2.5_, and reduced the level of oxidative damage in aPM_2.5_-exposed rats. Moreover, PD inhibited aPM_2.5_-induced inflammation response, as evidenced by downregulation of white blood cells in bronchoalveolar lavage fluid (BALF), inflammation-related lipids and proinflammation cytokines in lung. These results provide a practical means for self-protection against particulate air pollution.

Currently, environmental issues pose huge threats to public health, particularly the damage caused by fine particulate matter (aerodynamic diameter <2.5 μm; PM_2.5_). The high content of PM_2.5_ in the atmosphere is associated with morbidity and deleterious effects such as premature death among individuals due to lung disease, lung dysfunction and asthma exacerbation^1^,^2^,^3^,^4^. Although the Chinese government has taken a series of environment protection measures, the level of PM_2.5_ in this country is more than triple the acceptable annual level (i.e.,<35 μg/m^3^)^5^, highlighting the health risk posed by these particles^6^,^7^. In fact, PM_2.5_ has become an important health threat to people living in Mainland China^4^,^5^,^6^,^7^,^8^,^9^, and this problem is difficult to solve rapidly. Therefore, studies seeking to elucidate the mechanisms that promote disease progression contribute to identifying novel preventive and therapeutic targets and strategies.

The composition of PM_2.5_ mainly consists of sulfate, nitrate, nitrogen salt and heavy metals^10^. Mesoporous silica nanoparticles (MSN) are advantageous for harboring the major chemical components of PM_2.5_ because of their special structure with numerous small pores; these chemicals can adhere to the large inner surface of the pores^11^. Previous studies have reported that the primary injury associated with airborne particulate matter exposure is mainly localized in the respiratory system, causing inflammation and a reduction in lung function^1^,^2^,^9^, and the health effects is largely associated with the oxidative potential (OP) of PM_2.5_ particles^12^. However, attempts to understand the injury by PM_2.5_ particles have made only limited success and few studies have identified treatment regimens to prevent the tissue damage.

The objective of this study was to assess the effects of PM_2.5_ inhalation in healthy animals, and to identify medicinal materials that decrease the OP of PM_2.5_ particles and protect lung from PM_2.5_ attack. As the initial step, we created artificial PM_2.5_ (aPM_2.5_) particles based on the physical and chemical analysis of PM_2.5_ particles collected in the center of Beijing in 2014. Second, we investigated the effect of aPM_2.5_ on the respiratory system. Then, a screening test was performed to discover the compounds that might reduce OP on aPM_2.5_, through which we found polydatin (PD) was a good candidate to serve the purpose. PD is a resveratrol 3-O-D-glucoside isolated from *Polygonum cuspidatum*, a traditional Chinese medicine. PD, which is rich in grapes and red wine^13^, possesses anti-oxidant and anti-inflammatory activities^14^,^15^. Previous studies have demonstrated that PD treatment could ameliorate LPS-induced acute lung injury through reducing inflammation and apoptosis^16^. In addition, PD also reduced septic lung injury in mice through unregulated heme oxygenase (OH-)1 and inhibited proinflammation mediators in lung^17^. What presented below showed that PD could be a therapeutic agent to protect pulmonary impairment from air pollution.

## Results

### Construction of aPM_2.5_ based on the features of collected PM_2.5_ in Beijing

**Figure 1:**
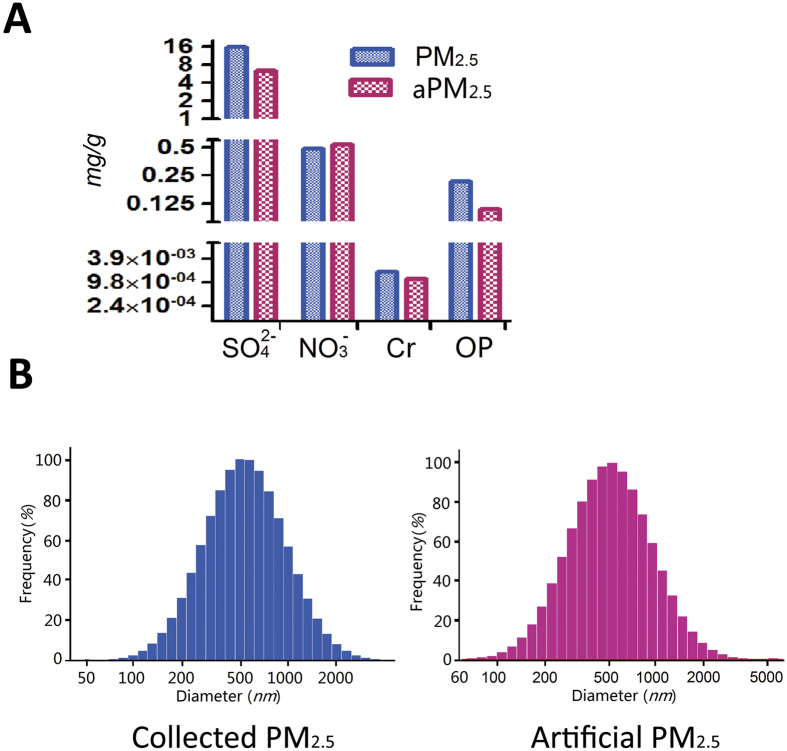
Chemical composition, size and OP value of the aPM_2.5_ compared with the collected PM_2.5_. (**A**) Chemical composition and OP values of the PM_2.5_ versus aPM_2.5_. (**B**) The size distribution of the PM_2.5_ and aPM_2.5_. All data are expressed as means.

The straightforward synthesizing process enables the scaled-up manufacture of MSN for inhalation exposure test assays^11^. Thus, aPM_2.5_ particles were prepared with MSN as the core and three chemical species loaded into the pores of MSN. Figure 1A shows that the aPM_2.5_ particles contained NO_3_^−^(mg/g), SO_4_^2−^(mg/g) and Cr (mg/g), with a mean diameter of 616 nm. Figure 1 shows the comparison of the compositions and mean diameters of the aPM_2.5_ with the collected PM_2.5_. The collected PM_2.5_ were 631 nm in diameter, identical to that of aPM_2.5_ (Fig. 1B), and uniform to the aPM_2.5_ in chemical components SO_4_^2−^, NO_3_^−^ and Cr (Fig. 1A). Moreover, the OP of the aPM_2.5_ was approximately 60% that of the collected PM_2.5_ (Fig. 1A). These results demonstrate that the aPM_2.5_ were similar to the PM_2.5_ in terms of chemical composition, diameter, and OP.

### PD reduces the OP of aPM_2.5_

**Figure 2:**
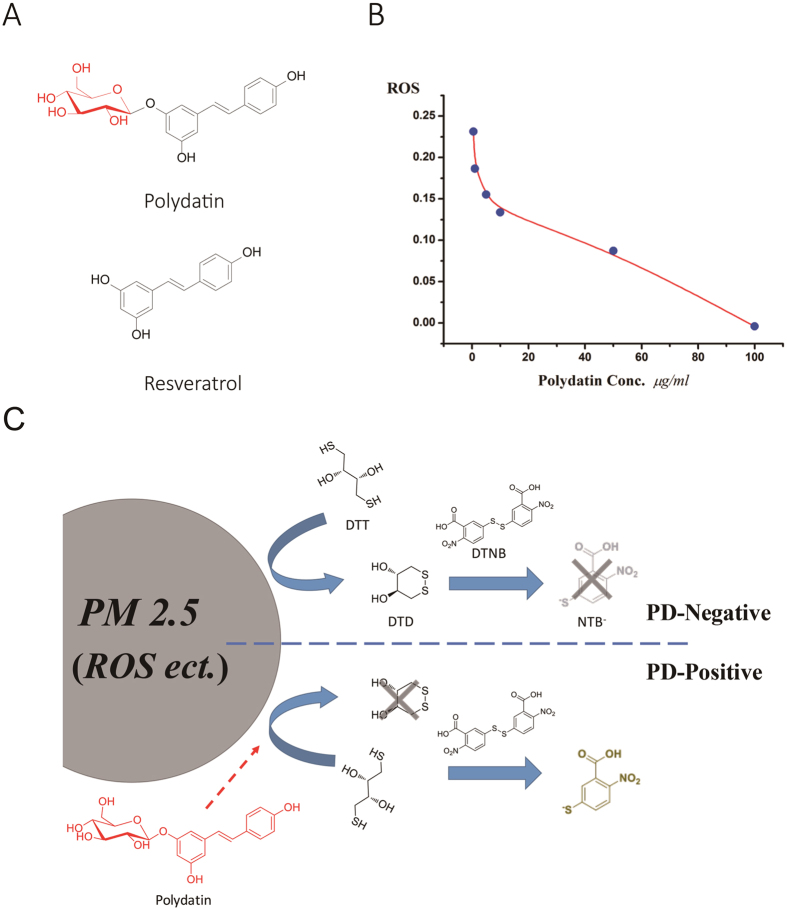
PD reduces the OP of aPM_2.5_. (**A**) Structures of resveratrol and PD. (**B**) PD reduced the OP of aPM_2.5_. (**C**) The putative chemical mechanism of OP reduction by PD. DTT: dithiothreitol; DTD: 1,2-dithiane-4,5-diol; DTNB: 5,5′-Dithiobis-(2-nitrobenzoic acid); NTB-: 5-thio-2-nitrobenzoic acid.

To screen compounds that are able to reduce oxidative stress of PM_2.5_, the dithiothreitol (DTT) assay was used. In our cell-free reaction system, PD (Fig. 2A; mw, 350) significantly reduced the OP of aPM_2.5_ in a dose-dependent manner (Fig. 2B). Therefore, we speculated that PD may help to reduce aPM_2.5_-induced damage in the respiratory system. The putative chemical mechanism for this effect is shown in Fig. 2C. Particulate matter (PM) is an oxidative and able to catalyze ROS production^18^. DTT was added into aPM_2.5_ to mimic reducing-materials in the body. In the absence of PD, DTT could be converted into DTD (1,2-dithiane-4,5-diol) which is not able to react with chromogenic agent DTNB (5,5’-Dithiobis-(2-nitrobenzoic acid)). Thus, no yellow product NTB^−^ (5-thio-2-nitrobenzoic acid) is generated. In the presence of PD which favorably reacts with ROS on aPM_2.5_, the conversion from DTT to DTD via oxidation was inhibited; the DTT mainly reacts with DTNB, producing NTB^−^, showing yellow color in the reaction. Thus, PD decreases the ability of aPM_2.5_ to catalyze ROS production, resulting in a reduced OP value on the aPM_2.5_.

### PD protects rats from the aPM_2.5_-induced lung function changes

The rats were exposed to aPM_2.5_ via inhalation at an average aPM_2.5_ concentration of 1.13 g/m^3^. The exposure time was 60 min per day and 7 days a week for 8 weeks. The equivalent aPM_2.5_ concentration to which the rats were exposed in the chamber was “normalized” over the 8-week period and was 47.1 mg/m^3^ after considering the non-exposed time^19^. Respiratory function was measured every 2 weeks during exposure.

**Figure 3:**
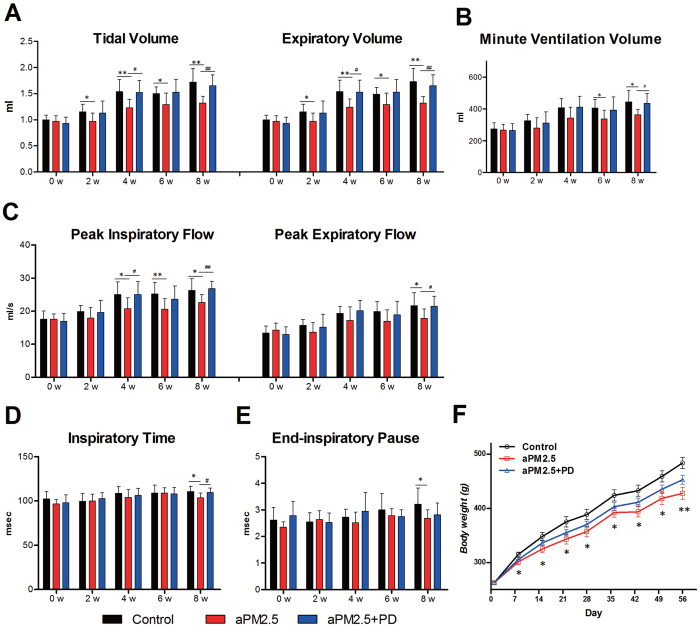
PD protects rats from aPM_2.5_ exposure. PD improves the decrease in pulmonary ventilation function caused by aPM_2.5_. (**A**) Tidal volume and expiratory volume and (**B**) minute ventilation volume (ml). (**C**) The peak inspiratory and expiratory flow (ml/s). (**D**) Inspiratory time and (**E**) end-inspiratory pause (msec) of conscious rats. Data are expressed as means ± SD (n = 8). *P  < 0.05, **P  < 0.01, versus the control group. ^#^*P * < 0.05, ^##^*P * < 0.01, versus the aPM_2.5_ group. (**F**) Body weight (g) development of rats during aPM_2.5_ exposure for 8 weeks. Data are expressed as the means ± SEM (n = 8). *P  < 0.05, **P  < 0.01, versus the control group.

The rats responded to aPM_2.5_ attack by showing time-dependent lung function reduction (Fig. 3A–E). Treating aPM_2.5_-exposed rats with PD (50 mg/kg, oral) for 8 weeks showed a significant protective effect against the aPM_2.5_-induced lung function changes (Fig. 3A–E). Exposure to aPM_2.5_ is associated with a significant reduction in tidal volume, expiratory volume and minute ventilation volume in the aPM_2.5_-exposed rats, in respect to that in the control group (Fig. 3A,B). In contrast, PD treatment reversed the effect on lung by aPM_2.5_ in all of these parameters above. Airway resistance increased after the rats were exposed to aPM_2.5_ from 4 to 8 weeks, as evidenced by the change in flow index of the ventilation function, including decreases in peak inspiratory flow and peak expiratory flow (Fig. 3C). However, after PD treatment, these indices rebounded, showing values similar to that of controls. The inspiratory time and end-inspiratory time of aPM_2.5_-exposed rats were decreased (Fig. 3D,E) as compared with the controls; however, the indices for rats in the aPM_2.5_ + PD group were increased. In addition, the results showed no significant effect of treatment on expiratory time, frequency, end-expiratory pause and Penh (not shown).

The body weight increase of the rats in the 8 weeks was 84.4% for the control group, 62.9% for aPM_2.5_ group and 72.2% for aPM_2.5_ + PD group. Apparently, the aPM_2.5_ particles caused significant growth inhibition (Fig. 3F, *P * < 0.05). Body weights of the aPM_2.5_ + PD group were higher than that of the aPM_2.5_ group, but not statistically significant (*P * > 0.05).

### PD ameliorates aPM_2.5_-induced oxidative damage in rats

**Figure 4:**
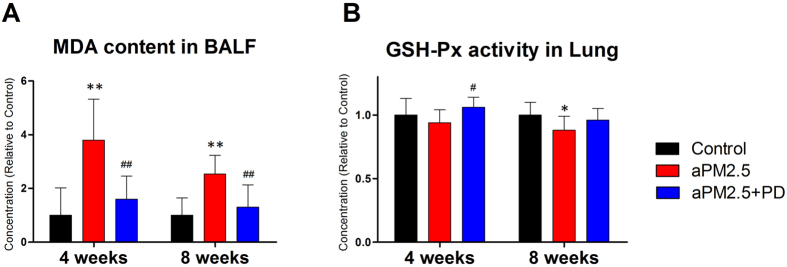
aPM_2.5_ induces oxidative damage in rats, whereas PD protects against these effects. (**A**) MDA levels in BALF. (**B**) GSH-Px in lung. The results are expressed as means ± SD (n = 8). *P  < 0.05 and **P  < 0.01, versus the control group; ^#^*P* < 0.05 and ^##^*P * < 0.01, versus the aPM_2.5_ group.

To determine whether aPM_2.5_ induces the formation of reactive oxygen species (ROS) in the lung and blood, we analyzed the malondialdehyde(MDA) level (a product of lipid peroxidation) and the activity of antioxidant glutathione peroxidase(GSH-Px). Particle exposure induced a significant increase in MDA in the BALF, reaching a 3.8-fold increase (*P * < 0.01) at 4 weeks and a 2.5–fold increase (*P * < 0.01) at 8 weeks as compared to the control; while PD treatment significantly decreased the MDA levels, reaching a 58% decrease at 4 weeks and a 48% decrease at 8 weeks relative to the aPM_2.5_ group (Fig. 4A, *P * < 0.01). The levels of lung GSH-Px measurement showed a reduction after exposure to aPM_2.5_, and PD treatment restored the lung GSH-Px levels (Fig. 4B). It appears that aPM_2.5_ induced oxidative damage to the lung, and PD was protective against the aPM_2.5_-caused damage.

### PD suppresses aPM_2.5_-induced inflammation in lung

**Figure 5:**
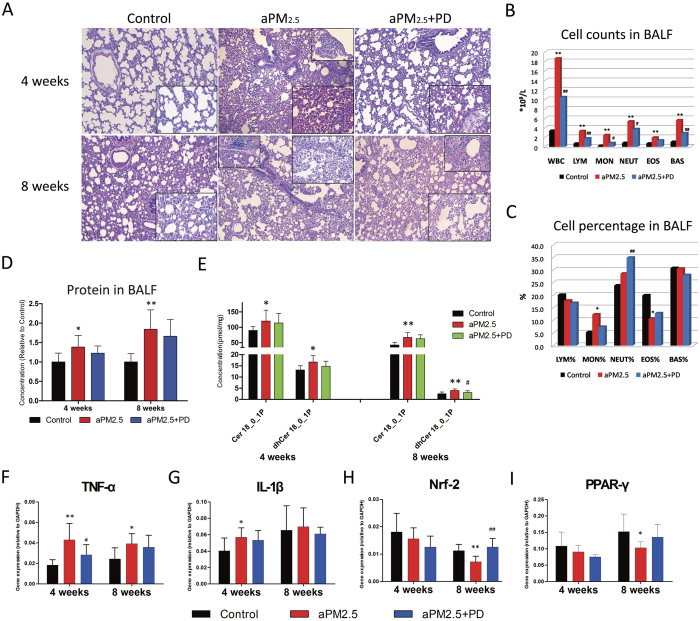
PD prevented aPM_2.5_-induced inflammation in the pulmonary interstitial space. (**A**) A histological analysis of lung injury in rats. Representative low-power ( × 100) and high-power (*inset*, × 400) H&E-stained lung sections after exposure for 4 and 8 weeks. (**B**) Cell counts and (**C**) cell percentages in BALF after exposure for 8 weeks. The results are expressed as means (n = 8). (**D**) Protein level in BALF. The concentrations are shown as relative to the control. (**E**) The change in the level of ceramides in the rat lung tissues. The results are expressed as means ± SD (n = 8). Relative gene expression of (**F**) tumor necrosis factor (TNF)-α, (**G**) interleukin (IL)1-β, (**H**) nuclear factor NF-E2-related factor 2 (Nrf2) and (**I**) peroxisome proliferator–activated receptor (PPAR-γ). Values are reported using the 2^−ΔCt^ method. Samples were normalized to GAPDH gene expression. The results are expressed as means ± SD (n = 8). *P < 0.05 and **P < 0.01, versus the control group; ^#^*P* < 0.05 and ^##^*P* < 0.01, versus the aPM_2.5_ group.

Histopathological examination (n = 8) showed consistent time-dependent changes in the lung after exposure to aPM_2.5_ (Fig. 5A). Upon reviewing the literature, we found that the aPM_2.5_ caused pathological damage in the rat lungs was similar to that by PM_2.5_ in humans^3^,^20^. Inhalation of aPM_2.5_ resulted in interstitial thickening and inflammatory cell infiltration, featured with infiltration of the macrophage and lymphocyte. After 4 weeks exposure, there were increased macrophages and lymphocytes in the lung tissue, significant exudation of inflammatory cells with alveolar wall thickening, and red blood cells scattered in the alveolar areas. The pathological damage was milder in the aPM_2.5_ plus PD group than that in the aPM_2.5_ group, with less wall thickening and decreased macrophage infiltration. After exposure for 8 weeks, the pathological damage in the aPM_2.5_-exposed rats aggravated, with a largely increased infiltration of macrophages and inflammatory cell around the alveolar wall, and thickness of local alveolar septae. However, reduced alveolar wall thickening and lung inflammation were observed in the group treated with PD for 8 weeks.

Accordingly, the BALF samples showed changes in the percentage and the absolute number of the cells (Fig. 5B). By week 8 of the experiment, significant increases in the total white blood cell (WBC), lymphocyte (LYM), monocyte (MON), neutrophil (NEUT), eosinophil (EOS) and basophil (BAS) counts were detected in the aPM_2.5_ group (Fig. 5B, *P* < 0.01), and the most pronounced increase was in the MON% (Fig. 5C, *P* < 0.05). However, PD treatment significantly deceased the number of WBC (*P * < 0.01), LYM (*P* < 0.01), MON (*P* < 0.05), NEUT (*P* < 0.05) and BAS (*P* < 0.01) counts in the BALF, as compared to the aPM_2.5_-exposed group (Fig. 5B). Reason for the increase of NEUT% remains unclear (Fig. 5C).

The BALF total protein (TP), as an indication of lung inflammation, increased by 1.39-fold (*P < *0.05) in 4 weeks and 1.84-fold (*P* < 0.01) in 8 weeks in the aPM_2.5_-exposed rats, in comparison with the controls, indicating a capillary leakage and alveolar barrier injury by aPM_2.5_ (Fig. 5D). PD treatment resulted in a decrease of the BALF protein, but not statistically significant (Fig. 5D).

Previous studies have shown that lipids are involved in the inflammation process^21^. We therefore examined the lipid profile and found that inflammation-related lipids were significantly different between the control and aPM_2.5_ groups in the lung. The lipid levels (Cer 18_0_1P and dhCer 18_0_1P) changed in the lung tissue after exposure to the aPM_2.5_ (Fig. 5E). In both short and long-term exposure to aPM_2.5_, the two lipids increased in lung tissue; and more increase of lipids was observed after long-term exposure to the aPM_2.5_ in respect to the short-term exposure group (Supplementary Figure 1), suggesting that long-term aPM_2.5_ exposure closely associates with lung inflammation. However, PD treatment neutralized the lipids in the lung tissue both for short- and long-term (*P* < 0.05) exposure groups. It appears that PD inhibits the inflammation provoked by aPM_2.5_.

To further characterize the *in vivo* inflammatory response, we evaluated the proinflammatory factors in the lung tissue. As compared to the control rats, TNF-α mRNA expression was 2.34-(*P* < 0.01) and 1.61-fold (*P* < 0.05) increased in the lung of the aPM_2.5_-exposed rats at 4 and 8 weeks, respectively (Fig. 5F). The mRNA expression of the IL-1β was also up-regulated in the aPM_2.5_ –treated rats at week 4 (*P* < 0.05) but not week 8 (*P* > 0.05, Fig. 5G). PD treatment inhibited aPM_2.5_-induced TNF-α and IL-1β mRNA expression, particularly at week 4, when TNF-α was decreased by 34% (*P* < 0.05, vs. aPM_2.5_ rats, Fig. 5F). For the rats subjected to prolonged exposure (8 weeks), PD administration restored Nrf-2 (*P* < 0.01) expression, which was suppressed by aPM_2.5_ (*P* < 0.01, Fig. 5H). PD also protected against the aPM_2.5_-induced PPAR-γ decrease, as shown in Figure 5I. Other inflammatory factors, including IL-6, ICAM-1 and MCP-1, were unaffected by aPM_2.5_ (*P* > 0.05, data not shown). These results indicate that PD treatment might decrease the OP on the surface of the aPM_2.5_ and suppressed pro-inflammatory cytokines during aPM_2.5_ dosing.

### Red wine is rich in PD

**Figure 6:**
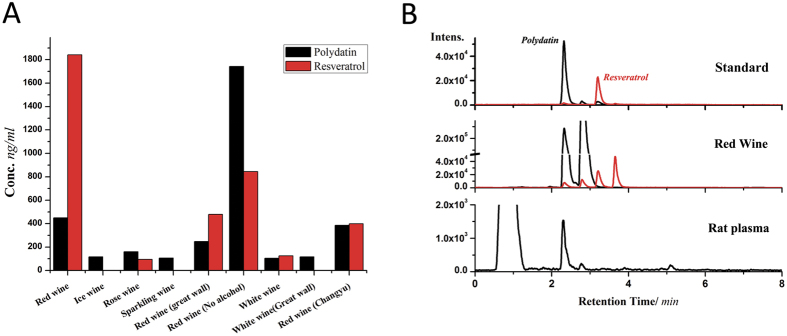
The PD content in wines and blood. (**A**) Levels of PD and resveratrol in wine. (**B**) Chromatogram of the PD in wine and rat blood samples.

To accelerate the application of PD in ameliorating airborne PM_2.5_-caused respiratory injury, the levels of PD and resveratrol in 9 types of wine and blood were examined using HPLC-MRM. As Fig. 6A shows, PD was detected in all 9 wines, and the concentrations varied from hundreds to thousands ng/ml. Red wine contained more PD than the others. PD was detectible in blood samples 24 hours after the oral administration of PD in rats (50 mg/kg); however, resveratrol was not (Fig. 6B), which suggests that PD is well adsorbed in the intestine and thoroughly distributed in the blood. Thus, the notable protective effects on the lungs can be attributed to PD, rather than resveratrol.

## Discussion

It has been reported that PM_2.5_ could cause lung injury, including aggravated asthma, decreased lung function, increased respiratory symptoms, which leads to higher mortality and hospitality^3^,^9^,^22^,^23^,^24^. The present study successfully created a multi-component aPM_2.5_. The injury by aPM_2.5_ has been demonstrated by showing decreased lung function, oxidation damage, pathological changes and inflammation response in lung tissue. The results were in consistent with that reported in human study^2^,^20^. The damage by PM_2.5_ is at least partially associated with OP on the particles. We found that PD, which is a natural compound in fruits like grapes, is an agent protecting lung damage from aPM_2.5_ exposure. This might be linked to its ability to reduce OP value of PM.

Quantitative analyses of the effects of air pollution on lung function are unsatisfactory because of the differences among the size and composition of particles^25^. The aPM_2.5_ used in the present study was created based on information of the real life PM_2.5_ in Beijing, and nanotechnology to provide a standardized exposure assessment. The chemicals that adhere onto PM_2.5_ can include ions (SO_4_^2−^, NH_4_^+^, and NO_3_^−^) and benzene rings and pollutant elements (Al, Pb, Fe, and Cr), and the percent of each varies^26^. We created aPM_2.5_ using MSNs as the particle core with typical ions and metals adhering to the core. The aPM_2.5_ was consistent with the collected PM_2.5_ obtained from Beijing in terms of its size, elements and OP values. Inhalation of aPM_2.5_ in rats caused function and tissue damage in lung, and the injury progressed in a time-dependent manner.

PM_2.5_ exposure is associated with decreases in lung function^9^,^21^,^25^,^26^. Our results showed that the breath flow and volume parameters decreased in animals after exposure to aPM_2.5_^23^,^27^, supporting the hypothesis that PM_2.5_ exposure might reduce lung function. As other traditional parameters, such as P:F ratio or Sa0_2_, are important in evaluating lung function, these indications will be included in further investigation.

ROS generation is a key event associated with PM_2.5_-induced lung injury and linked to inflammation^19^,^28^,^29^,^30^. In this study, aPM_2.5_ particle exposure caused a gradual accumulation of oxidative damage, particularly in the lungs. Oxidative stress induced lung dysfunction was accompanied increases in protein leak. In addition, oxidative stress related biomarkers were examined to evaluate the oxidation-related damage. In the present study, alteration of MDA and GSH-Px was detected. The aPM_2.5_ exposure inhibited Nrf-2 expression in the lungs; Nrf-2 is a gene product related to oxidative stress that controls cellular adaptation to oxidants via transcriptional activation through antioxidant responsive elements (ARE)^28^,^29^,^30^,^31^.

Oxidative stress causes inflammation in tissue^31^,^32^. At each time point after particle exposure, we observed an initial robust lung inflammatory response, including inflammatory cell infiltration, increased histological injury and BALF protein elevation^20^. The generation of ceramide as an intermediate of sphingolipid metabolism might cause pulmonary diseases triggered by cigarette smoke and carbon nanoparticles^21^,^33^. In fact, particle-exposure leads to the accumulation of ceramides in lipid rafts, and this effect is consistently associated with ceramide in humans^34^. Our results showed the alteration of ceramides in the development of lung oxidative stress and inflammation in response to aPM_2.5_. A previous study found that particle-exposure increased the secretion of IL-1β and TNF-α^35^, which is consistent with our results. PPAR-γ is a ligand-activated transcription factor that regulates lipid metabolism and the immune response. In our rat model, aPM_2.5_ inhibited PPAR-γ function and might have contributed to the pathogenesis of lung injury^36^.

The OP of atmospheric PM_2.5_ is related to airway oxidative stress and inflammation^1^,^12^,^37^,^38^, which suggests that depleting the OP of these particles might be effective in reducing PM_2.5_-caused damage. We showed that PD, the bioactive ingredient in *Polygonum cuspidatum root*, has a potent antioxidant activity^39^. Previous research has shown that PD is absorbable in intestine via an active mechanism and might be metabolized into resveratrol in the blood^40^. Therefore, we anticipate that PD might have bioactivity similar to that of resveratrol, a well-known compound with an anti-oxidization effect. Indeed, PD reduced the OP of PM_2.5_ *in vitro*. The current animal model revealed that PD (50 mg/kg/d, oral, for 28 and 56 days) significantly protected lung function and pathologic changes from aPM_2.5_. PD treatment significantly decreased MDA levels but increased GSH-Px activity. Moreover, PD inhibited aPM_2.5_-induced inflammation, reducing infiltration, maintaining alveoli structure, preventing ceramide chaos and inhibiting inflammatory cytokines (TNF-α and IL-1β) production in the lung^41^. These defense mechanisms might involve the regulation of the Nrf-2 and PPAR-γ pathways^15^,^42^. For BAL protein production and weight change the PD protection was observed but not at significant level. There could be at least two reasons. First, the two parameters might not be sensitive to the reduction of OP value; and second, more PD might be needed to show the effect on these two indications.

The Chinese government launched the National Action Plan on Air Pollution Control in 2013, which requires a reduction in PM_2.5_ in major urban areas^43^. Furthermore, urgent self-protection from PM_2.5_-caused respiratory system damage has become a serious public health concern. The aPM_2.5_ created in the study could simulate the characteristics and effects of the real PM_2.5_, providing a useful research material for future investigation against air pollution. Although human studies are needed, the present study shows, for the first time, that PD may represent a way to protect personal health before the PM_2.5_ level returns to normal.

## Materials and Methods

### Preparation of aPM_2.5_

MSNs were synthesized via the Stöber method^44^ using tetraethyl orthosilicate (TEOS, J&K Chemical Ltd., Beijing, China) as a silicon source and CTAB (J&K Chemical Ltd., Peking, China) as the template. Three chemical species, H_2_SO_4_, NH_4_NO_3_, and K_2_Cr_2_O_7_, were loaded into the pores of MSNs using the impregnation method. MSN (100 g each) was added to 1 L of ethanol solution containing 0.04 mg/ml of H_2_SO_4_, 0.04 mg/ml of NH_4_NO_3_, and 1.6 × 10^−6^ mg/ml of K_2_Cr_2_O_7_. Magnetic stirring was performed at room temperature for 24 h to maximize the loading efficiency. Then, the chemical species-loaded MSNs (aPM_2.5_) were collected via centrifugation, washed twice with ethanol, and dried under a vacuum. To determine the size of the aPM_2.5_, a particle powder (2.0 mg) was resuspended and ultrasonicated in 10 ml of water and then measured with a NICOMP 380 ZLS Particle Sizer (USA).

### Analysis of bio-samples by HPLC-MRM

The analysis of PD and resveratrol was performed using an Agilent 1260 infinity HPLC system and a 6490 triple quadrupole mass spectrometer. Bio-samples were extracted as previously reported^45^. The 5-μl extraction volume was injected onto the column and gradient eluted into MS. The MRM parameters were as follows: resveratrol = 227− > 185 (20.0 eV) and PD = 389− > 227 (20.0 eV).

HPLC-MRM was also applied to the bio-samples for ceramide analysis. Ceramides were extracted from plasma and tissues as reported previously^46^. Statistical analyses were conducted based on the data acquired using the Agilent 1200 HPLC system and a 6410 triple quadrupole mass spectrometer.

### Reduction of aPM_2.5_ OP by PD

A total of 2 mg aPM_2.5_ was extracted with 1 ml methanol with sonication for 20 min. After 15 min of centrifugation at 13,000 rpm, the supernatant was collected and dried. The residues were reconditioned with 1 ml PB (0.1 M, pH 7.4). A volume of 700 μl solution was mixed with 200 μl PD solution and 100 μl DTT (1 mM). The mixture was incubated at 37 °C for 30 min. A volume of 25 μL DTNB (20 mM in methanol) was added and measured at 412 nm.

### Animals

Eight-week-old male specific pathogen-free Sprague–Dawley (SD) rats were obtained from HFK Bioscience Co., Ltd. (Beijing, China). After acclimation for 2 weeks, the rats were housed in groups in an environmental-controlled barrier-sustained animal room in Peking-Union Pharma animal center, which achieved AAALAC international accreditation. The animals were supplied with standard commercial diet and drinking water *ad libitum*. Environmental controls for the animal room were set to a temperature of 22–24 °C, a relative humidity of 50–60% and a 12 h light/12 h dark cycle. The Institutional Animal Ethics Committee of New Drug Safety Evaluation Center at the Institute of Materia Medical (Beijing, China) approved the study before it began and all the animal studies were carried out in accordance with the approved guidelines and regulation.

### Inhalation exposure to aPM_2.5_

A total of 48 SD rats were randomly assigned to the following three groups of sixteen: (i) control group, (ii) aPM_2.5_ group and (iii) aPM_2.5_ + PD group. One hour before exposure, rats in the aPM_2.5_ + PD group were given PD (50 mg/kg, i.g.), whereas rats in the other two groups were given distilled water (10 ml/kg). Animals in the control group were exposed to filtered air, whereas rats in the aPM_2.5_ and aPM_2.5_ + PD groups were exposed to particles using a nose-only inhalation system that was under slight pressure due to a particle flow of 1 ml/min and an internal flow of 0.8 m^3^/h. Then, the animals were randomly fixed on the aerosol generator to ensure the balance and consistency of the inhaled PM aerosol or fresh air. Rats were exposed for 60 min/day, 7 days/week using a Hope-med 8052 automatic inhalation device (Hope-med Industry and Trade Co., Ltd., Tianjin, China). Body weight was measured at the beginning of exposure, twice per week during exposure, and before necropsy, and 8 rats from each group were sacrificed at 4 and 8 weeks.

We measured the concentration of the aPM_2.5_ aerosol weekly with a gravimetric filter analysis^47^,^48^. The aPM_2.5_ were collected on glass fiber filters (40 mm, 0.8-μm pore) and loaded into polypropylene filter cassettes using a dust sampler (FC-1B BMILP, Beijing, China) at a flow rate of 2 L/min. The filters were pre- and post-weighed in a temperature- and humidity-controlled weighing room using a BSA224S-CW microbalance (Sartorius, Germany). The mean concentration (C) of the aPM_2.5_ obtained by calculation was 1.13 g/m^3^.

The particle size distribution of the resuspended particles in our exposure system was measured using an *Aerodynamic Particle Sizer* APS (3321, TSI, MN, USA), and the mass median aerodynamic diameter (MMAD) was 1.17 μm. The GSD was 1.80.

The inhalable fraction (IF) and alveolar deposition fraction (DF) were calculated for rats using Multiple Path Particle Deposition (MPPD V2.1, 2009)^48^,^49^. Specifically, the MMAD, GSD and aerosol concentrations from the rat exposures were obtained. The density of the aPM_2.5_ was approximately 0.125 g/m^3^. The results were generated as a regional fraction of the entire lung. Based on these assumptions and other input data^46^ (Table 1), the IF was 0.9941, and the DF was 0.0383. These calculation results are consistent with the experimental data of Peter and Philip^49^,^50^. After exposed for 4 and 8 weeks, the deposition dosages of the aPM_2.5_ in the alveolar region were 593.6 μg/day and 603.9 μg/day, respectively. The steady state particle mass burdens in the alveolar region were 16.3 mg and 33.0 mg, respectively.

### Pulmonary function measurement

Lung function was assessed as described previously^51^. Respiratory function was measured using a pulmonary function testing system (Emka Technologies, Paris, France), and the raw data were converted using specialized analysis software (IOX 2.9.4.32, Emka Technologies). Briefly, conscious rats were placed in plethysmography chambers for more than 15 minutes, eliminate the data animals adapt to their environments or sleep. The measured indices included inspiratory time, expiratory time, peak inspiratory flow, peak expiratory flow, tidal volume, expiratory volume, relaxation time, minute respiratory volume, end-inspiratory/expiratory pause and respiratory frequency.

### BALF collection

At each time point, eight rats in each group were anesthetized with pentobarbital (40 mg/kg, i.p.) 24 hours after exposure to aPM_2.5_ or filtered air. BALF was collected by slowly instilling and withdrawing 12 ml of cold saline into the left lung 3 times through tracheal intubation^52^. The BALF was centrifuged (1,500 rpm for 10 min). The cell-free supernatants were assayed for different analyses, and the cell pellets were resuspended in 200 μl WBC dilution buffer for cell counts and classification. Lung tissues were removed and stored at −80 °C until use.

### Histology

Following BALF collection, the lung right lobe specimens and nasopharyngeal mucosa were removed and weighed, fixed with 4% paraformaldehyde, embedded in paraffin and sectioned at 4-μm thickness using an RM2145 microtome (LEICA, Germany). The sections were stained with hematoxylin and eosin (H&E) and observed under a light microscope (LEICA, Germany).

### Determination of ceramides

High-performance liquid chromatography coupled with tandem mass spectrometry (HPLC-MS/MS) was performed using an Agilent 6410B Triple Quad mass spectrometer (Agilent Technologies Inc., Santa Clara, CA) comprising a triple quadrupole MS analyzer equipped with an electrospray ionization interface and an Agilent 1200 RRLC system (HPLC-MS/MS). The HPLC-MS/MS methodology used in this study was described in our previous report.

### Quantitative reverse transcriptase real-time polymerase chain reaction (qRT-PCR)

Total RNA was extracted from the right lung tissues of SD rats using the TRIzol^®^ Plus RNA Purification Kit (Invitrogen, USA). Briefly, tissue samples were homogenized in 1 ml TRIzol Reagent per 50 mg tissue using a tissue homogenizer, and the quantity and purity of the total RNA were evaluated using a nanodrop 2000c UV spectrophotometry (Thermo Scientific, USA) at 260 and 280 nm. Using the Power SYBR^®^ Green RNA-to-CT™ 1-Step Kit (Thermo Scientific, USA), 10 ng of total RNA was transcribed into cDNA. The cDNA products were then used as the template for PCR amplification, and the detection of the specific gene expression was performed using an ABI 7500 Fast instrument (Life Technologies Inc., Carlsbad, CA, USA). The sequences of the primers used are listed in Table 2. Expression differences were calculated using the 2^−ΔCt^ method^53^. GAPDH was used as an endogenous reference.

### Measurement of TP, MDA and GSH-Px

Assay kits for TP, MDA and GSH-Px were provided by Jiancheng Bioengineering Institute (Nanjing, China). The contents were measured according to the manufacturer’s instructions. TP levels in the BALF was expressed as μg/ml. MDA levels in the BALF were expressed as nmol/ml. GSH-Px in the lung was expressed as U/mg protein. The final data were expressed as ratios compared with controls to correct for systematic errors at different times.

### Data analyses

Heterogeneity of variance among groups was first examined by F-tests, and further analyses for the difference between groups were done using unpaired 2-tail Student’s t-test. Significance was defined as *P* < 0.05. Values in the graphs are presented as means and standard deviations (SD) (or standard error (SEM)). Microsoft Excel 2013 (Microsoft Corporation) and the GraphPad Prism5 (GraphPad Software) were used for data management, statistical analysis and graph generation.

## Additional Information

**How to cite this article**: Yan, X.-D. *et al*. Polydatin protects the respiratory system from PM_2.5_ exposure. *Sci. Rep.* **7**, 40030; doi: 10.1038/srep40030 (2017).

**Publisher's note:** Springer Nature remains neutral with regard to jurisdictional claims in published maps and institutional affiliations.

## Supplementary Material

Supplementary Information

## Figures and Tables

**Table 1:** Model parameter settings in MPPD.

Model Parameter	Settings
Airway Morphometry	Species: Rats
	FRC = 4.0 ml
	URT volume = 0.42 ml
Particle Properties	Density = 0.125 g/cm^3^
	MMAD = 1.17 μm (Single)
	GSD = 1.80 μm
	Nanoparticle model & inhalability adjustment
Constant exposure	Gravity = 981 cm/s^2^
	Body orientation: On stomach
	Aerosol concentration = 1,130 mg/m^3^
	Breathing Scenario: Nasal
Deposition & Clearance	Rat clearance rate: 0.00105/days
	Exposure time: 1 h/day, 7 days/week for 4 or 8 weeks

**Table 2:** PCR primer sequences.

Gene Name	Sense (5′------3′)	Anti-sense (5′------3′)
TNF-α	CCACCACGCTCTTCTGTCTAC	AGGGTCTGGGCCATGGAACT
IL-1β	TACCTATGTCTTGCCCGTGGAG	ATCATCCCACGAGTCACAGAGG
Nrf-2	CAGTGCTGCTGTGCACGAAT	AGCCTCTAATCGGCTTGAAT
PPAR-γ	CCCACCAACTTCGGAATCAG	GGAATGGGAGTGGTCATCCA
GAPDH	AACCTGCCAAGTATGATGACATCA	ACAACTTCGGCGTCCTCTGTTGGA
