# Supplementary material for: Polydatin protects the respiratory system from PM2.5 exposure
Source: Sci Rep. 2017 Jan 9;7:40030. doi: 10.1038/srep40030 (PMC5220290; doi:10.1038/srep40030)
Supplement: Supplementary Information [file srep40030-s1.doc]

**Supplementary information**

**Polydatin protects the respiratory system from PM_2.5_ exposure**

Xiao-Dan Yan^#^, Qi-Ming Wang^#^, Cai Tie^#^, Hong-Tao Jin, Yan-Xing Han, Jin-Lan Zhang, Xiao-Ming Yu, Qi Hou, Piao-Piao Zhang, Ai-Ping Wang, Pei-Cheng Zhang*, Zhonggao Gao*, and Jian-Dong Jiang*

State Key Laboratory of Bioactive Substances and Function, Institute of Materia Medica, Chinese Academy of Medical Sciences, Peking Union Medical College, Beijing 100050, China

^#^These authors contributed equally to this work.

*Corresponding authors

Correspondence and requests for materials should be addressed to J.-D.J. (E-mail: jiang.jdong@163.com).

Mailing address: Institute of Materia Medica, Chinese Academy of Medical Sciences, Peking Union Medical College, No. 2 Nan Wei Street, West District, Beijing 100050, China

Phone: 86-010-83160005

Fax: 86-010-83160005

E-mail: [jiang.jdong@163.com](mailto:jiang.jdong@163.com)

**Supplementary Figure 1.** The changes of lipids levels in lung tissues in the rat exposed to aPM_2.5_. Rats exposed to aPM_2.5_ for (A) 4 weeks and (B) 8 weeks. The results are expressed as the mean ±
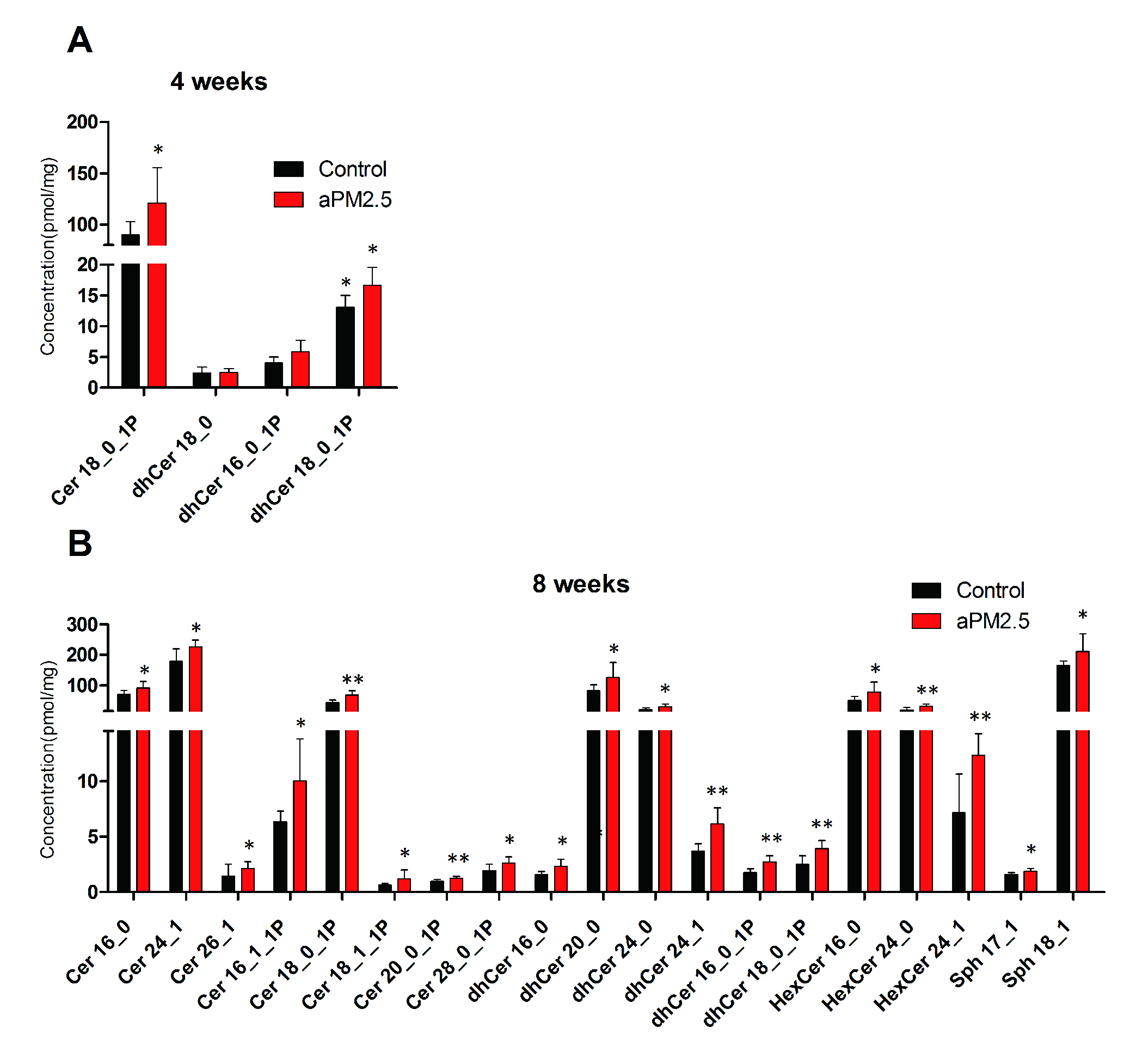
SD (n=8). *P<0.05 and **P<0.01, significantly different with respect to the control group.
